# Prevalence of Dermatophytic Infection and the Spectrum of Dermatophytes in Patients Attending a Tertiary Hospital in Addis Ababa, Ethiopia

**DOI:** 10.1155/2015/653419

**Published:** 2015-09-13

**Authors:** Gebreabiezgi Teklebirhan, Adane Bitew

**Affiliations:** ^1^Tikur Anbessa Hospital, College of Health Sciences, Addis Ababa University, P.O. Box 1176, Addis Ababa, Ethiopia; ^2^Department of Medical Laboratory Sciences, College of Health Sciences, Addis Ababa University, P.O. Box 1176, Addis Ababa, Ethiopia

## Abstract

*Background*. Dermatophytosis is common worldwide and continues to increase.* Objective*. This study was undertaken to determine the prevalence of dermatophytosis and the spectrum of ringworm fungi in patients attending a tertiary hospital.* Methods*. Samples were collected from 305 patients. A portion of each sample was examined microscopically and the remaining portion of each sample was cultured onto plates of Sabouraud's dextrose agar containing chloramphenicol with and without cycloheximide. Dermatophyte isolates were identified by studying macroscopic and microscopic characteristics of their colonies.* Result*. Of 305 samples, fungi were detected in 166 (54.4%) by KOH of which 95 were dermatophytes while 242 (79.4%) samples were culture positive of which 130 isolates were dermatophytes. Among dermatophyte isolates* T. violaceum* was the most common (37.7%) cause of infection. Tinea unguium was the predominant clinical manifestation accounting for 51.1% of the cases. Patients with age group 25–44 and 45–64 years were more affected.* T. violaceum *was the most common pathogen in tinea unguium and tinea capitis, whereas* T. mentagrophytes *was the most common pathogen in tinea pedis. *Conclusion*. Further intensive epidemiological studies of ring worm fungus induced dermatophytosis which have public health significance are needed.

## 1. Introduction

Superficial mycoses are among the most frequent forms of human infections, affecting more than 20–25% of the world's population [1]. They are predominantly caused by a group of closely related keratinophilic mycelia fungi (dermatophytes) in the genera of* Trichophyton*,* Microsporum*, and* Epidermophyton*. These groups of fungi invade the stratum corneum of the skin or other keratinized tissues derived from the epidermis such as hair and nails [2, 3].

Although dermatophytosis is considered to be a trivial disease, the psychological effects of the disease are highly considerable and because of its high morbidity, it is a costly disease in terms of loss of working days and treatment [4].

Dermatophytes have been recorded all over the world but with variation in distribution, incidence, epidemiology, and target hosts from one location to another. Geographic location, climate (temperature, humidity, wind, etc.), overcrowding, health care, immigration, environmental hygiene culture, and socioeconomic conditions have been incriminated as major factors for these variations [1, 5].

According to Havlickova et al. [1] and Ilkit [6], the prevalence of dermatophytosis has significantly reduced in many developed nations of the world compared to the developing ones due to improved social, economic, health care, and hygiene practice factors, evident in the former.

Studies that investigated the prevalence of dermatophytosis and its etiologic agents in Ethiopia are few and most of them were carried out on a specific section of a population, that is, school children [7–10], and these studies may not be a true representation of the overall disease pattern of the country. Ethiopia being a developing nation located in the tropic with wet humid climate appears to fell into the category of regions with high prevalence of dermatophytosis. Furthermore, Ethiopia as one of the developing countries, socioeconomic constraints and other common prevalent health issues have led to a low awareness of dermatophytosis by physicians and general population and hence conducting further studies to know the actual magnitude of dermatophytosis as well as the spectrum of its etiological agents among the general population is of the highest priority.

## 2. Materials and Methods

### 2.1. Sample Collection

A total of 305 clinical samples were collected from patient visiting the Dermatology Department of Tikur Anbessa Teaching Hospital, College of Health Sciences Addis Ababa University. The samples were collected from September 2014 to October 2015. Before collecting the sample the infected area was cleaned with 70% (v/v) ethanol. Then skin and finger nail samples were collected by scrapping of lesion with sterile blade and dull broken hairs from the margin of scalp lesion with forceps and transferred to sterile folded papers. Each of these papers was appropriately labeled with the age, sex, date of collection, code of a patient, and location of infection and taken to the Microbiology Laboratory of the Department of Medical Laboratory Science, College of Health Sciences within the date of collection.

### 2.2. Culture and Microscopic Examination

A portion of each sample was mounted in a drop of an aqueous solution of 10%  (w/v) potassium hydroxide (KOH) on a clean microscopic slide. After 5 minutes of mounting, the preparation was examined under low (×10) and high (×40) power magnification for the presence of fungal elements. The remaining portion of each clinical sample was cultured irrespective of the negative or positive direct microscopic examination results onto plates of Sabouraud's dextrose agar containing chloramphenicol with and without cycloheximide (Oxoid, Basingstoke, England) which were prepared according to the manufacture's instruction. All inoculated plates were then incubated at inverted position for 4–6 weeks at 25–30°C aerobically. Culture plates were examined twice a week for any fungal growth. Colonies suspected of dermatophytes were subcultured into potato dextrose agar (Oxoid, Basingstoke, England) for the production of spores. Cultures of dermatophytes were identified by examining macroscopic and microscopic characteristics of their colony. Texture, rate of growth, topography, and pigmentation of the front and the reverse side of the culture were employed for the macroscopic identification. Microscopic identification of mold isolates was performed by placing pieces of a colony from SDA and/or PDA to clean microscopic slide and staining with lactophenol cotton blue. After placing a cover slip, each preparation was observed microscopically. Urea agar (Oxoid, Basingstoke, England) was used in the differentiation of* Trichophyton tonsurans*,* Trichophyton violaceum*, and* Trichophyton rubrum*. All ethical considerations and obligations were duly addressed and the study was conducted after the approval of the ethical committee of Internal Department Review board and obtaining written consent from study subjects.

## 3. Result

In the present study a total of 305 clinical samples were collected from suspected cases of dermatophytosis of which 97 (31.8%) were from males and 208 (68.2%) from females. The ages of study subjects ranged from 1 year to 80 years with a mean age of 26 years. The details regarding clinical manifestation and sex of study subjects were given in Table 1. Tinea unguium was the predominant clinical manifestation accounting for 51.1% of the cases of which 119 (76.3%) were females and 37 (23.7%) males. This was followed by tinea corporis and tinea capitis accounting for 20.0% and 10.8% of the cases, respectively.

Fungal elements were detected in 166 (54.4%) of clinical samples by KOH wet mount of which 95 were dermatophytes while 242 (79.4%) clinical samples were culture positive of which 130 isolates were dermatophytes. Further identification of dermatophytic fungi showed the presence of* Trichophyton mentagrophytes*,* Trichophyton rubrum*,* Trichophyton tonsurans*,* Trichophyton soudanense*,* Trichophyton violaceum*,* Trichophyton verrucosum*,* Trichophyton schoenleinii*,* Epidermophyton floccosum*,* Microsporum nanum*,* and Microsporum audouinii*. Among all the dermatophyte isolates* T*.* violaceum* was the most common (37.7%) cause of infection, followed by* T*.* mentagrophytes* (17.7%) and* T*.* tonsurans* (16.9%), whereas* M*.* nanum* (0.8%) was the least common.

Clinical manifestation in relation to age group depicted that patients with age group 25–44 and 45–64 years were equally affected each accounting for 32.5% of the cases followed by age group 14–24 years accounting for 21.3%. Tinea unguium was found to be more in patients of age group 25–44 years and tinea pedis in patients of age group 45–64 years. Tinea capitis was common in patients of age group of 1–14 years (Table 2).

According to species frequency in different areas of involvement,* T*.* violaceum* was the most common pathogen in tinea unguium and tinea capitis, whereas* T*.* mentagrophytes* was the most common pathogen in tinea pedis and tinea manum (Table 3).

The distribution of dermatophytes in relation to different age groups was variable.* T*.* violaceum* and* T*.* mentagrophytes* were the dominant pathogens in age group 45–64 accounting for 42.9% and 34.8%, respectively.* T*.* tonsurans* was the most frequently isolated dermatophyte in age group 25–44 accounting for 31.8% (Table 4).

## 4. Discussion

Dermatophytic infections are more prevalent in the developing world and the infection is increasing in this part of the world. Despite this fact, studies on dermatophyte infections in Ethiopia are scanty and the results of these studies may not be a true representation of the overall disease pattern of the country. The present study attempted to determine the dermatophyte infections in patients attending a tertiary teaching hospital in Addis Ababa, Ethiopia.

Of the 305 clinical samples collected from patients with cases of suspected dermatophytosis referred to the Department of Dermatology, Tikur Anbessa Hospital, College of Health Sciences Addis Ababa University in the period of September 2014 to October 2015, dermatophytes were detected in 95 (31.1%) samples by KOH wet mount and 130 (42.6%) samples were culture positive for dermatophytes. The present prevalence rate of culture proven dermatophytic infection was relatively low, compared to earlier local surveys (Ethiopia) among school children with rates between 33% and 73% [9, 10]. A prevalence rate of KOH proven dermatophyte infections ranging from 53.1% to 100% and a prevalence rate of culture proven dermatophytic infections ranging from 52.2% to 67.1% have been reported by Kannan et al. [11] and by Ellabib and Khalifa [12]. Disparity in the prevalence rates of dermatophytosis in different studies could result from differences in the lifestyle, socioeconomic conditions, risk factors associated with study subjects, and environmental factors of study area [1, 3].

The present study showed that more females were affected by dermatophytes than males, with female-male ratio being 2.2 : 1. Earlier studies also indicated a higher prevalence of dermatophytes in females compared to males [13–16]. Meanwhile some other earlier studies recorded a higher prevalence of dermatophytes in males than females [17, 18].

The predominant clinical manifestations of dermatophytosis vary considerably in different studies reported in literature. In a study conducted in India, tinea corporis (35.4%) was the predominant clinical condition followed by tinea cruris (16.8%) and tinea capitis (16.7%) [13]. Similar study conducted in Iran between March 2005 and March 2007 by Rassai et al. [14] revealed that tinea cruris and tinea corporis were the most common clinical manifestation. A 7-year (1997–2003) survey of dermatophytoses in Crete, Greece, conducted by Maraki et al. [15] revealed that tinea unguium was the predominant clinical manifestation. A study carried out by Devliotou-Panagiotidou et al. [16] between 1981 and 1990 in Greece depicted that tinea pedis was the most frequent clinical manifestation. Adefemi et al. [17] reported tinea capitis as a predominant clinical manifestation. In our study, tinea unguium was the dominant clinical manifestation involving 51.1% of the total cases of dermatophytosis, similar to many other reports [15, 18]. Tinea capitis was the second clinical manifestation accounting for 61 (20%) of dermatophytosis as has been observed in other studies [17, 19, 20]. Tinea corporis was the third common clinical presentation accounting for 33 (10.8%) and this clinical manifestation has been reported as a dominant clinical manifestation by earlier similar studies [12–14].

In the present study persons of all age groups were susceptible to dermatophytosis but it appeared to be more common in adults of age group 25–44 and 45–64 years each accounting for 32.5% of the cases as they are physically active outdoors. Our finding in this regard was compatible with the findings of others [7, 9]. As universally reported by most of the workers, tinea capitis is an infection of childhood. In the present study a total of 61 patients with tinea capitis, and 21 patients were in age group of 1–14. Similar results were reported by earlier researches [21, 22]. The changing pattern of hormones after puberty [23] and production of inadequate amounts of inhibitory fatty acids before puberty [24] are responsible for a decrease of tinea capitis with age. On the other hand, tinea unguium was more frequent in the elderly population with an age group of 25–64. Reduced growth rate of the ungual plate, an increase in trauma rates, poor peripheral circulation, and inability to maintain good foot care could be attributed to this [25]. On the other hand tinea pedis was a dominant clinical manifestation in age group 45–64 years which was in agreement with the findings of Lange et al. and Caputo et al. [26, 27]. Of the total number of 130 dermatophytes isolates in the present study 72.3% was accounted by* T*.* violaceum*,* T*.* mentagrophytes*, and* T*.* tonsurans*. Among the three dominant species,* T*.* violaceum* accounted for 37.69% of the total isolates and our finding was compatible to studies conducted in Ethiopia [7–10], several other African countries [26–28], and several Asian countries [25, 29, 30].* T*.* violaceum* has been reported as one of the endemic dermatophytes in the horn of Africa and Asia by Ameen [3]. Although we have no immediate reason for small number cases of* T*.* schoenleinii* and* T*.* tonsurans* as one of the dominate dermatophytes in the present study as opposed to previous studies in East Africa, the heterogeneity in the distribution of dermatophytosis, their etiologic agents, and the predominating clinical manifestation patterns in different parts of the world have been attributed to factors of geographic location, climate, overcrowding, health care, immigration, environmental hygiene culture, and socioeconomic conditions [1, 5].

## 5. Conclusion

This study has revealed that the prevalence of microscopic and culture confirmed dermatophytic infections in the study subjects was high. The present study has also depicted that tinea unguium was the dominant clinical manifestation involving 51.1% of the total cases of dermatophytosis. Of the total number of 130 dermatophyte isolates 72.3% was accounted for by* T*.* violaceum*,* T*.* mentagrophytes*, and* T*.* tonsurans*. Among the three dominant species,* T*.* violaceum* accounted for 37.69% of the total isolates. Because of the psychological effects and high morbidity in terms of loss of working days and treatment dermatophytic infection is a public health problem. Therefore, to obtain a true representation of the overall disease pattern of the country more such types of studies should be conducted.

## Figures and Tables

**Table 1:** Frequency and percentage distributions of clinical manifestations in relation to sex (*n* = 305).

Clinical manifestation	Sex		Total
	Male	Female	
Tinea capitis	24 (39.3%)	37 (60.7%)	61 (20%)
Tinea corporis	7 (21.2%)	26 (78.8%)	33 (10.8%)
Tinea cruris	4 (100.0%)	0 (0.0%)	4 (1.3%)
Tinea unguium	37 (23.7%)	119 (76.3%)	156 (51.1%)
Tinea pedis	6 (40.0%)	9 (60.0%)	15 (4.9%)
Tinea faciei	9 (45.0%)	11 (55.0%)	20 (6.6%)
Tinea manum	10 (62.5%)	6 (37.5%)	16 (5.2%)
Total	97 (31.8%)	208 (68.2%)	305 (100%)

**Table 2:** Frequency of clinical manifestation in different age groups (*n* = 305).

Site	Age groups					Total
	1–14	15–24	25–44	45–64	≥65	
Tinea capitis	21 (34.4%)	14 (23.0%)	13 (21.3%)	13 (21.3%)	0 (0.0%)	61 (19.9%)
Tinea corporis	4 (12.1%)	5 (15.2%)	16 (48.5%)	6 (18.2%)	2 (6.1%)	33 (10.8%)
Tinea cruris	0 (0.0%)	0 (0.0%)	0 (0.0%)	2 (50.0%)	2 (50.0%)	4 (1.3%)
Tinea unguium	9 (5.8%)	42 (26.9%)	54 (34.6%)	48 (30.8%)	3 (1.9%)	156 (51.0%)
Tinea pedis	1 (6.7%)	1 (6.7%)	5 (33.3%)	8 (53.3%)	0 (0.0%)	15 (4.9%)
Tinea faciei	0 (0.0%)	3 (15.0%)	5 (25.0%)	12 (60.0%)	0 (0.0%)	20 (6.5%)
Tinea manum	0 (0.0%)	0 (0.0%)	6 (37.5%)	10 (62.5%)	0 (0.0%)	16 (5.2%)
Total	35 (11.5%)	65 (21.3%)	99 (32.5%)	99 (32.5%)	7 (2.3%)	305 (100%)

**Table 3:** Prevalence pattern of dermatophytes across clinical manifestations (*n* = 130).

Fungal isolates	Clinical manifestations							Total
	Tinea capitis	Tinea corporis	Tinea cruris	Tinea ungium	Tinea pedis	Tinea faciei	Tinea manum	
*T*. *violaceum*	17 (44.7%)	4 (21.0%)	0 (0.0%)	19 (38.0%)	1 (16.7%)	6 (66.7%)	2 (28.6%)	49 (37.7%)
*E*. *floccosum*	0 (0.0%)	2 (10.5%)	0 (0.0%)	0 (0.0%)	1 (16.7%)	1 (11.1%)	0 (0.0%)	4 (3.1%)
*T*. *soudanense*	1 (2.6%)	2 (10.5%)	0 (0.0%)	2 (4.0%)	0 (0.0%)	0 (0.0%)	0 (0.0%)	5 (3.8%)
*T*. *mentagrophytes*	5 (13.1%)	1 (5.3%)	1 (100%)	7 (14.0%)	3 (50.0%)	1 (11.1%)	5 (71.4%)	23 (17.7%)
*T*. *tonsurans*	7 (18.4%)	4 (21.0%)	0 (0.0%)	9 (18.0%)	0 (0.0%)	1 (11.1%)	0 (0.0%)	21 (16.0%)
*T*. *rubrum*	4 (10.5%)	2 (10.5%)	0 (0.0%)	4 (8.0%)	1 (16.7%)	0 (0.0%)	0 (0.0%)	11 (8.5%)
*T*. *schoenleinii*	3 (7.9%)	3 (15.8%)	0 (0.0%)	2 (4.0%)	0 (00%)	0 (0.0%)	0 (0.0%)	8 (6.0%)
*T*. *verrucosum*	0 (0.0%)	1 (5.3%)	0 (0.0%)	3 (6.0%)	0 (0.0%)	0 (0.0%)	0 (0.0%)	4 (3.0%)
*M*. *audouinii*	1 (2.6%)	0 (0.0%)	0 (0.0%)	3 (6.0%)	0 (0.0%)	0 (0.0%)	0 (0.0%)	4 (3.0%)
*M*. *nanum*	0 (0.0%)	0 (0.0%)	0 (0.0%)	1 (2.0%)	0 (0.0%)	0 (0.0%)	0 (0.0%)	1 (0.8%)
Total	38 (29.2%)	19 (14.6%)	1 (0.8%)	50 (38.5%)	6 (4.6%)	9 (6.9%)	7 (5.4%)	130 (100%)

**Table 4:** Distribution of dermatophytes in relation to different age categories (*n* = 130).

Species	Age categories in years					Total
	1–14	15–24	25–44	45–64	≥65	
*T*. *violaceum*	7 (14.3%)	12 (24.5%)	9 (18.4%)	21 (42.9%)	0 (0.0%)	49 (37.7%)
*T*. *mentagrophytes*	4 (17.4%)	4 (17.4%)	6 (26.1%)	8 (34.8%)	1 (4.3%)	23 (17.7%)
*T*. *tonsurans*	6 (27.3%)	5 (22.7%)	7 (31.8%)	4 (18.2%)	0 (0.0%)	22 (16.9%)
*T*. *rubrum*	1 (10.0%)	2 (20.0%)	5 (50.0%)	2 (20.0%)	0 (0.0%)	10 (7.7%)
*T*. *soudanense*	0 (0.0%)	0 (0.0%)	4 (80.0%)	0 (0.0%)	1 (20.0%)	5 (3.8%)
*T*. *schoenleinii*	2 (25.0%)	5 (62.5%)	1 (12.5%)	0 (0.0%)	0 (0.0%)	8 (6.2%)
*T*. *verrucosum*	0 (0.0%)	2 (50.0%)	1 (25.0%)	1 (25.0%)	0 (0.0%)	4 (3.1%)
*M*. *audouinii*	1 (25.0%)	2 (50.0%)	0 (0.0%)	1 (25.0%)	0 (0.0%)	4 (3.1%)
*M*. *nanum*	0 (0.0%)	0 (0.0%)	0 (0.0%)	0 (0.0%)	1 (100%)	1 (0.8%)
*E*. *floccosum*	0 (0.0%)	0 (0.0%)	3 (75.0%)	0 (0.0%)	1 (25.0%)	4 (3.1%)
Total	21 (16.1%)	32 (24.6%)	36 (27.7%)	38 (29.2%)	4 (3.1%)	130 (100%)
